# Changes in the nutrient composition of top-selling packaged foods and beverages in Colombia between 2016 to 2021

**DOI:** 10.3389/fnut.2025.1534195

**Published:** 2025-02-11

**Authors:** Luis Carlos Forero, Luis F. Gómez, Mercedes Mora-Plazas, María Parra-Murillo, Sara Toquica, Lindsey Smith Taillie

**Affiliations:** ^1^Departamento de Nutrición Humana, Universidad Nacional de Colombia, Bogotá, Colombia; ^2^Facultad de Medicina, Pontificia Universidad Javeriana, Hospital Universitario San Ignacio, Bogotá, Colombia; ^3^Department of Health Behavior, Gillings School of Global Public Health, University of North Carolina at Chapel Hill, Chapel Hill, NC, United States; ^4^Universidad Nacional de Colombia, Bogotá, Colombia; ^5^Department of Nutrition, Gillings School of Public Health, University of North Carolina at Chapel Hill, Chapel Hill, NC, United States

**Keywords:** noncommunicable disease prevention, Latin America, food policy, food labeling, food supply, Colombia

## Abstract

**Introduction:**

In 2022, the Colombian government approved a law requiring by 2024 the use of Front of Package octagonal warning labels in food products with an excess of nutrients of concern for chronic disease, including sodium, sugar, saturated fat, and trans-fat, as well as non-nutritive sweeteners (NNS). In addition, the government began 2023 by applying a 10% tax on sugar-sweetened beverages and foods that also had warning labels for sodium, sugar and saturated fat. This tax increased to 15% in 2024 and to $20% in 2025. While a previous study examined the changes in the nutritional composition of beverage and food products offered in Colombia between 2016 and 2018, it is necessary to update this information to understand whether the industry anticipated labeling and tax regulations by reformulating products.

**Methods:**

This study carried out a comparison of the content of selected nutrients of concern and non-nutritive sweeteners (NNS) from the nutritional panels of 164 matched pairs of packaged foods and beverages from the top selling brands in Colombia in 2021 compared to 2016. McNemar’s test for paired data was used to evaluate changes in the proportion of each of the critical nutrients and NNS to be regulated.

**Results:**

There were no significant changes in sodium, saturated fat, trans fat, or free sugar in foods or beverages prior to policy implementation. However, there was a significant increase in the presence of NNS in beverages, during this pre-policy period from 32 to 59%.

**Conclusion:**

These findings suggest that changes in nutrient composition of packaged foods and beverages had been marginal before the implementation of the tax and warning labeling laws.

## 1 Introduction

Unhealthy dietary patterns, such as the consumption of ultra-processed foods and beverages (UPFs), are a leading risk factor for death and a significant burden of disease in Colombia (1). The UPFs are packaged or ready to eat products characterized by being high in energy density, sodium, added sugar, and saturated fats, while being low in dietary fiber, protein, vitamins, and minerals (2, 3). Consistent evidence shows that regular consumption of UPFs increases the risk of obesity, hypertension, cardiovascular diseases, and type 2 diabetes, among other noncommunicable diseases (NCDs) (4, 5).

In Colombia, the consumption of UPFs, particulary those high in sugar, sodium, and saturated fat, has increased concomitantly with the prevalence of overweight, obesity, and other diet related NCDs. According to the National Nutritional Situation Survey (ENSIN) of Colombia, between 2010 and 2015, the prevalence of overweight and obesity increased by 5.2 percentage points in adults (18–64 years), 2.4 in adolescents (13–17 years) and 5.6 in school children (5–12 years) (6, 7). In addition, the PURE cohort study reported a high prevalence of hypertension in Colombian adults aged 35–70 (37.4%) (8).

To address this growing concern about diet-related NCDs, in 2021, the Ministry of Health of Colombia implemented the mandatory use of circular warning labels in food products with an excess of nutrients of concern, including sodium, sugar, saturated fat. In 2022, this policy was replaced by a system of octagonal warning labels on foods with excessive sodium, sugar, saturated fat and trans fat, as well as non-nutritive sweeteners (NNS), required by 2024. In November 2023 the Colombian government also implemented a 10% tax on sugary drinks and foods high in sugar, sodium and saturated fats, with final 20% tax levels and nutrient thresholds to be implemented in 2025. These policies are likely to prompt reformulation, as companies are incentivized to cut sugar, sodium, saturated fat, and other nutrients of concern to avoid being subject to the tax or warning labels. However, evidence about anticipatory reformulation is mixed. For example, in Chile, it was found that there was little or no reformulation by the industry before to the implementation of a regulation (9).

One major question relating to these measures is the extent to which they incentivize the reduction of nutrients of concern to avoid regulation (10–12). The industry promotes reformulation as a process that makes food products healthier for consumers, contributing to the prevention of NCDs related to food and dietary patterns (9). In this sense, the main objective of reformulation is changing the composition of UPFs without modifying dietary patterns (10). For example, sodium reduction is a cost-effective intervention to diminish the negative impact of high blood pressure (13). However, the nutrient changes in the reformulation process may occur with no significant reduction in caloric density (14). In addition, industry may replace sugar with NNS to maintain sweetness, despite reports that these substitutes are not recommended (15, 16).

This is particularly of interest to understand in Colombia, because while the octagonal warning label policy includes a label on NNS, the tax policy did not include products containing NNS within the scope of the tax. Thus, it is relevant to understand the extent to which the food industry still chose to replace sugar with NNS to avoid the tax, even if NNS was required to be labeled.

More generally, there is growing evidence that UPFs increase the risk of weight gain beyond the excess of nutrients of concern, further suggesting minimal health gains from reformulation of these products (17). While a previous study examined the changes in nutrition composition in UPFs offered in Colombia in the period 2016–2018 (18), it is useful to understand whether there was any reformulation in subsequent years, given the anticipation of new regulations. Therefore, this study aimed to assess the changes in the content of nutrients of concern and NNS defined by the Colombian regulation in the packaged foods and beverages between 2016 and 2021.

## 2 Methods

This study was conducted from two subsets of packaged foods and beverages offered in Colombia in 2016 and 2021. The criteria for selecting the products to be collected were that they were packaged offered in selected supermarkets and that they had Nutrition Facts Panel (NFP) data, ingredient list and barcode. The 2016 collection (*n* = 6,708) was conducted between August and November and covered all packaged products offered in the five supermarket chains with the highest sales in Bogota, the largest and most commercial city in the country. The selection of supermarkets was based on the information registered in Euromonitor database (19). Trained research assistants photographed products and entered information on nutrients and ingredients into the REDCap (Research Electronic Data Capture) (20).

The 2021 collection (*n* = 1,115) aimed to select the five best-selling food brands in Colombia, also identified by the Euromonitor database and the most frequently consumed packaged foods (Approximately 80%) reported in the ENSIN of Colombia (7). Because of the challenges of collecting packaged food data during the pandemic, the 2021 data collection was conducted by using secondary data on products from the Global Mintel New Product Database (50% of products). The remaining 50% of top-selling products not available in Mintel were collected via photographs acquired in supermarkets in Bogota and Medellín, using a similar protocol as in 2016. This collection was carried out prior to the implementation of the octagonal warning label.

**Figure 1:**
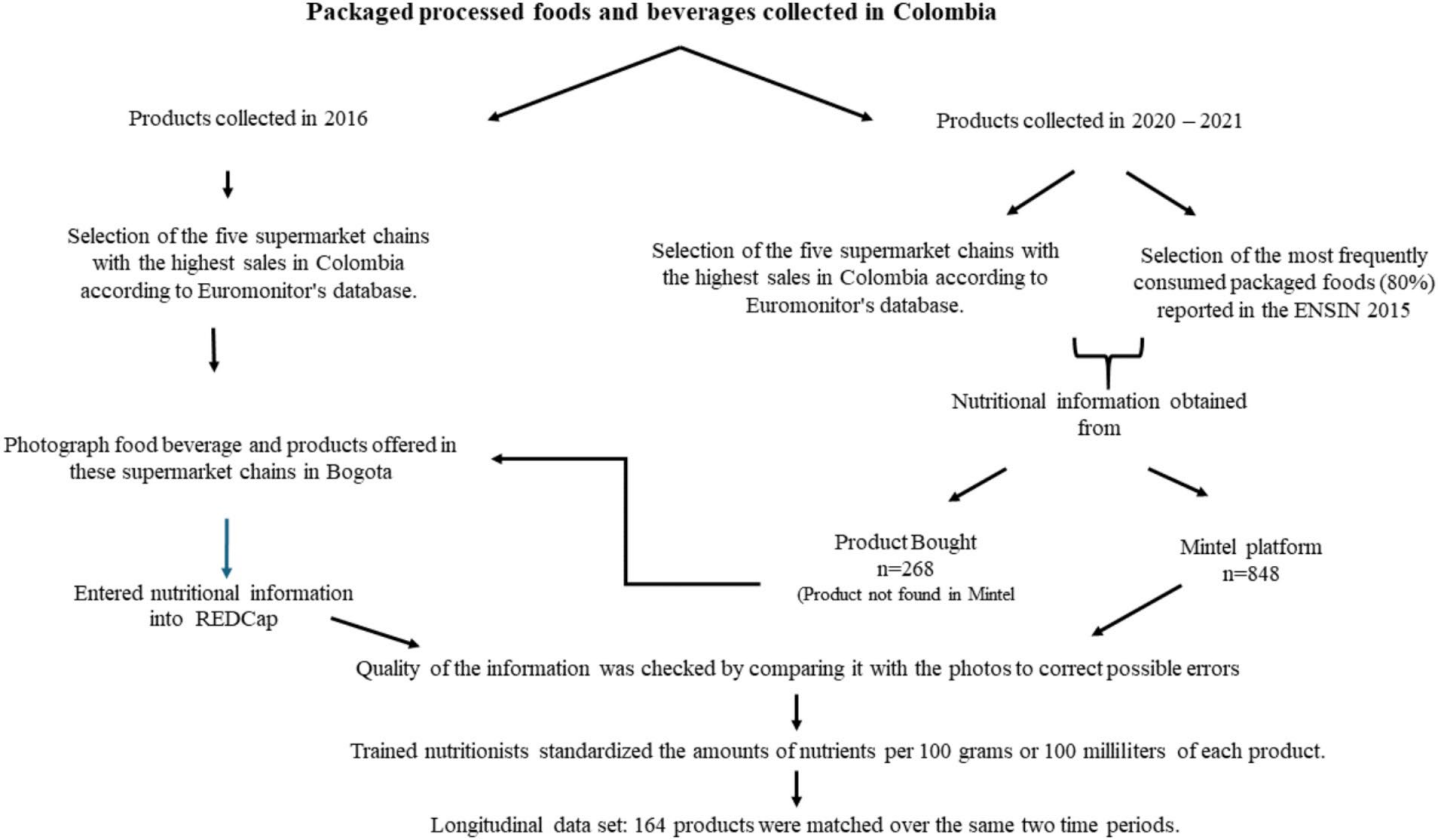
Packaged processed foods and beverages collected in Colombia.

In this study, the data collected in 2016 and 2021 were based on the photographic methods proposed by Kanter et al. (21) which were previously used in similar studies in Chile and Colombia (18, 22). The information from the (NFP) data, the ingredients list and the package details, such as barcodes were recorded and entered in REDCap. The data collected from the Mintel platform provided the photos of the products with the nutritional information required for the study and allowed them to be downloaded in Excel format. Once the data were entered in REDCap, and downloaded in Excel from the Mintel platform, the quality of the information was checked by comparing it with the photos to correct possible errors. Trained nutritionists standardized the amounts of nutrients per 100 grams or 100 milliliters of each product (Figure 1).

To create a longitudinal dataset (i.e., identify the same products in both time periods), we matched the product barcode, name, and brand or manufacturer. Thus, if an identical product was available in both 2016 and 2021, they were considered a matched pair. In cases where the product was found to be repeated two or more times for the same year, the first in the list was selected to avoid generating several matching pairs of the same product.

This study utilized a nutrient profile based on the Colombian regulation mandating the octagonal warning labels on packaged food and beverages containing excessive nutrients of concern (free sugar, sodium, saturated fats, trans fats) or any amount of non-nutritive sweeteners (NNS). This system applies the criteria from the nutrient profile model of the Pan American Health Organization (PAHO) Nutrient Profile Model (NPM) (22). However, the Colombian system does not include total fat and, conversely contains an additional and stricter sodium criterion (see Table 1). According to this regulation, products may have as many as five labels.

**Table 1:** Colombian nutrition model criteria for identifying processed and ultra-processed products excessive in sodium, free sugars, saturated fat, trans fat, and presence of non-nutritive sweeteners (NNS).

Nutrient	Solids and semi-solids	Liquids
Sodium	≥1 mg/kcal and/or ≥ 300 mg/100 g	≥1 mg/kcal (for NNS, non-Non-energy alcoholic beverages: ≥40 mg /100 mL)
Free sugars	≥10% of total kcal	≥10% of total kcal
Saturated fats	≥10% of total kcal	≥10% of total kcal
Trans-fats	≥1% of total kcal	≥1% of total kcal
NNS	Any amount of NNS	Any amount of NNS

Foods are classified by the PAHO NPM as unprocessed or minimally processed (fresh fish, tee, coffee, bottled water), culinary ingredients (edible oils, honey), processed food products (cheese, yogurt, tomato extract or concentrates, bread and bakery products), and ultra-processed products (packaged snacks, soft drinks, candies, instant soups and others).

Some foods were exempt from this assessment as they were excluded from warning label regulation and/or taxation. For example: foods and beverage for special use, such as baby food (infant formulas), and foods for special medical purposes; foods used as raw materials for industry and secondary ingredients that are not sold directly to the consumer; foods packaged in materials of natural origin and typical artisanal foods; hydrating drinks for athletes was exempted from warning label, but not for taxation.

In cases where the content of free sugars has not been declared on food labels, the PAHO Expert Advisory Group of the NPM presents a method to estimate the amount of free sugars based on a factor according to the information provided on the grams of total sugars (23). For example, if the manufacturer of a yogurt or milk reports that it contains sugars in the list of ingredients, and the NFP states that it contains 24 grams of total sugar and no free sugar, the PAHO NPM establishes for this case a factor of 0.5. The total sugar will then be multiplied by 0.5 to obtain the amount of free sugar, which would be calculated as 12 grams (24 g total sugar x 0.5).

For consistency, products were classified as food when the manufacturer declared their content in the package in grams (g) and were classified as beverages when the manufacturer declared their content in milliliters (mL). All dairy drinks reported in grams were considered solid food (include yogurt, kumis, oat flavored milk) and analyzed in “Dairy products”.

Powdered products were reconstituted according to package instructions, when caloric intake came exclusively from carbohydrates, sugars, or NNS and were classified as beverages (mL). Some products did not mention the reconstitution volume on their packaging, so their reconstitution was estimated at 200 mL per serving, and then nutrient densities were calculated of reconstituted product in water were calculated. Soup concentrates were not reconstituted.

### 2.1 Statistical analysis

The amounts of nutrients of concern (saturated fat, trans fat, free sugar, and sodium) per 100 g/mL were compared for the same products in 2016 and 2021. In addition, the proportions of those with at least one warning label and the presence of NNS were compared, considering the Colombian regulation.

For continuous variables, median and interquartile range (IQR) were estimated since the data were not normally distributed (Shapiro–Wilk test). To evaluate changes in nutrients by year, the Wilcoxon signed rank test was used as a nonparametric test to compare the median rank of two related samples (paired data). McNemar’s test for paired data was used to evaluate changes in the proportion of each of the critical nutrients and NNS to be regulated products.

The database was organized, reviewed, and filtered in Microsoft Excel and statistical processing and analysis was performed in Stata version 17. Significance alpha value was set at 0.05.

## 3 Results

A total of 164 matched pairs were found in the 2016 and 2021 dataset and included in the longitudinal analyses, 86.6% (*n* = 142) were food and 13.4% (*n* = 22) were beverages (Table 2). In general, no substantial differences were observed in the saturated fat, trans-fat, free sugar or sodium content for all products, foods, or beverages.

**Table 2:** Median (IQR) quantity per 100 g/mL of selected nutrients of concern in 2016 and 2021 from a longitudinal of food and beverage products^a^.

Category	Sat fat (g)		Trans fat (g)		Free sugar (g)		Sodium (mg)	
Year	2016	2021	2016	2021	2016	2021	2016	2021
All, *n* = 164	1.3	1.3	0.0	0.0	4.7	3.8	126.7	80.0
	(6.7)	(6.7)	(0.0)	(0.0)	(9.4)	(8.1)	(546.9)	(457.1)
Food, *n* = 142	1.7	1.6	0.0	0.0	3.9	3.8	300.0	241.7
	(8.6)	(8.0)	(0.0)	(0.0)	(12.0)	(11.1)	(629.2)	(465.0)
Beverage, *n* = 22	0.0	0.0	0.0	0.0	5.5	4.1	14.8	18.1
	(0.0)	(0.0)	(0.0)	(0.0)	(5.3)	(5.0)	(16.7)	(18.8)

a Parenthetical value represents the interquartile range (IQR). Mentioning no statistical differences.

**Figure 2:**
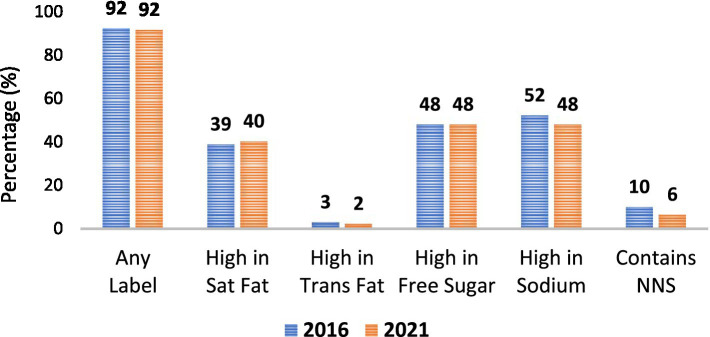
Solid foods that would be regulated under the Colombian nutrition model by year.

For food (Figure 2), 92% of products would carry at least one warning label in 2016 and 2021. There is no difference in the percentage of foods that would carry labels for saturated fat, trans fat, free sugar, sodium or presence of NNS. Across years, the highest proportion of “high in” products occurred for sugar and sodium, while few foods contained trans-fat (2–3%) or NNS (6–10%).

**Figure 3:**
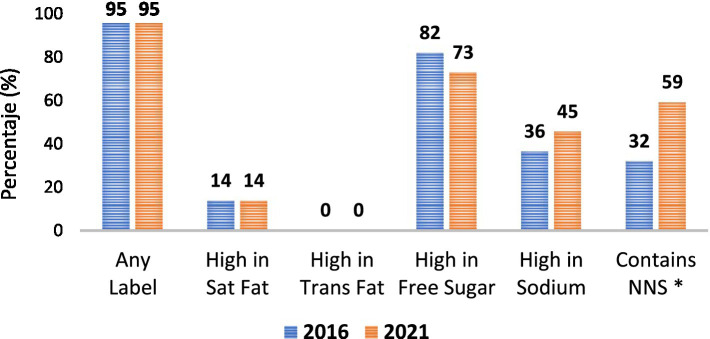
Beverages foods that are regulated under the Colombian nutrition model by year. *P*-values < 0.05 are considered statistically significant and are denoted by an *.

For beverages (Figure 3), 95% of products would carry at least one warning label in 2016 and 2021, with no differences across time, nor any differences for the percent of beverages that would carry labels for free sugar, sodium, saturated fat, or trans-fat. However, there was a significant increase in the percent of products containing NNS from 32 to 59% (0.031). In beverages, across years, the proportion of “high in” was highest for sugar, followed by sodium in 2016 and NNS in 2021.

## 4 Discussion

The results of this study provide information about the reformulation of top-selling packaged foods and beverages in Colombia in the period 2016 to 2021. Overall, the findings indicate the absence of substantial differences in saturated fats, trans fats, sodium and free sugar contents. There was a significant increase in the presence of NNS in beverages.

These results are similar to those found in a previous Colombian study (18) and suggest that the reformulation of processed and ultra-processed products was marginal before the implementation of the warning labeling law. This is also consistent with research conducted in Chile before the implementation of its labeling policy, which similarly found little to no anticipatory reformulation (9). One possibility in the case of Colombia is that reformulation may have occurred after 2021 and before November of 2023 when the tax law began to be implemented, or June of 2024 when the octagonal warning label law was finally enforced. Future research examining shorter periods within this implementation period (between 2022 and 2024) would be useful to understand how the industry responded to the laws during this period.

In general, our study found that a very high percentage of Colombian products would be considered high in nutrients of concern according to the law’s nutrient thresholds and thus would be required to carry at least one warning label (92% of foods and 95% of beverages). It is relevant to assess and understand the changes in food composition during the 2022–2024 transition period and after the implementation of the regulation.

The increase of NNS in beverages has been observed in other studies conducted in Latin American countries in the context of policy evaluation. For example, Peruvian research found an increase in NNS in beverages after implementation of the regulation (24). In Chile, the percentage of products containing NNS increased after the implementation of the regulation from 72% in 2015 to 82.6% in 2017 (25). In Chile and Peru, companies were likely replacing sugar with NNS to avoid the warning labels on sugar (these countries do not have a warning label on NNS). In Colombia, the law requires a warning label on NNS and sugar, which would in theory disincentivize reformulation. However, the sugary drinks tax law includes only beverages with sugar, not with NNS, which still provides a strong incentive to substitute sugar with NNS. It is relevant to consider this incentive to add NNS, due to recent reports that mention that NNS is not recommended for consumption by children and pregnant women (15, 16).

In addition, since the products analyzed in this study represent the five most sold brands in Colombia according to Euromonitor, the NNS reformulation found in this study could serve as a proxy for UPF consumption and changes in dietary patterns (26). The use of NNS in beverages and foods represents a reductionist approach in which product reformulation focuses on the sweet taste property to sell these products and does not consider the negative effects that this reformulation may have on health and the environment (27).

This study highlights the need for continued monitoring and research to inform effective public health strategies. The observed trends provide a foundation for future investigations, aiming to unravel the intricacies of dietary patterns and their impact on overall health and well-being.

This study has several limitations. First, the reduced number of products included in the analysis does not allow to conduct a robust examination of the entire spectrum of food and beverage subcategories. Second, matching pairs identified by barcodes was not possible, since if manufacturers modify the codes of reformulated products. Finally, this analysis did not capture changes in the Colombian food supply due to the introduction of new products and the exit of old products from the market.

## 5 Conclusion

This study did not find significant changes in the nutrients of concern during the period of analysis. However, there was an increase in the presence of NNS in beverages. The percentage of exceeding regulatory thresholds for at least one warning label exceeded 90% for both foods and drinks.

## Data Availability

The raw data supporting the conclusions of this article will be made available by the authors, without undue reservation.

## References

[1] Institute for Health Metrics and Evaluation. Global burden of disease study. (2019). Available at: https://vizhub.healthdata.org/gbd-compare/ (Accessed July 14, 2023)

[2] Monteiro CA Cannon G Levy RB Moubarac J-C Louzada ML Rauber F. Ultra-processed foods: what they are and how to identify them. Public Health Nutr. (2019) 22:936–41. doi: 10.1017/S1368980018003762, PMID: 30744710 PMC10260459

[3] Houshialsadat Z Cediel G Sattamini I Scrinis G Machado P. Ultra-processed foods, dietary diversity and micronutrient intakes in the Australian population. Eur J Nutr. (2024) 63:135–44. doi: 10.1007/s00394-023-03245-2 37798558 PMC10798929

[4] Wang M Du X Huang W Xu Y. Ultra-processed foods consumption increases the risk of hypertension in adults: a systematic review and Meta-analysis. Am J Hypertens. (2022) 35:892–901. doi: 10.1093/ajh/hpac069 35750049

[5] Barbosa SS Sousa LCM de D Pimentel JB Evangelista K Lyra C. A systematic review on processed/ultra-processed foods and arterial hypertension in adults and older people. Nutrients. (2022) 14:1215. doi: 10.3390/nu14061215, PMID: 35334872 PMC8955286

[6] Ministerio de la Protección Social. National Survey of the nutritional situation in Colombia 2010 ENSIN. 1st ed. ICBF, editor. Bogotá D.C., Colombia. (2011). 1–509 p.

[7] Minsalud. National Survey of the Nutritional Situation ENSIN (2015). 2nd ed. ICBF. Bogotá D.C., Colombia (2020). 1–336.

[8] Lamelas P Diaz R Orlandini A Avezum A Oliveira G Mattos A. Prevalence, awareness, treatment and control of hypertension in rural and urban communities in Latin American countries. J Hypertens. (2019) 37:1813–21. doi: 10.1097/HJH.0000000000002108, PMID: 30964825

[9] Kanter R Reyes M Vandevijvere S Swinburn B Corvalán C. Anticipatory effects of the implementation of the Chilean law of food labeling and advertising on food and beverage product reformulation. Obes Rev. (2019) 20:129–40. doi: 10.1111/obr.12870 31245920

[10] Gressier M Swinburn B Frost G Segal AB Sassi F. What is the impact of food reformulation on individuals’ behaviour, nutrient intakes and health status? A systematic review of empirical evidence. Obes Rev. (2021) 22:e13139. doi: 10.1111/obr.13139 33022095

[11] Scrinis G Monteiro CA. Ultra-processed foods and the limits of product reformulation. Public Health Nutr. (2018) 21:247–52. doi: 10.1017/S1368980017001392 28703086 PMC10261094

[12] Federici C Detzel P Petracca F Dainelli L Fattore G. The impact of food reformulation on nutrient intakes and health, a systematic review of modelling studies. BMC Nutr. (2019) 5:2. doi: 10.1186/s40795-018-0263-6 32153917 PMC7050744

[13] Barberio AM Sumar N Trieu K Lorenzetti DL Tarasuk V Webster J. Population-level interventions in government jurisdictions for dietary sodium reduction: a Cochrane review. Int J Epidemiol. (2017) 46:1551–405. doi: 10.1093/ije/dyw361 28204481 PMC5837542

[14] O’Mahony S O’Donovan CB Collins N Burke K Doyle G Gibney ER. Reformulation of processed yogurt and breakfast cereals over time: a scoping review. Int J Environ Res Public Health. (2023) 20:3322. doi: 10.3390/ijerph20043322 36834017 PMC9964677

[15] Sharma A Amarnath S Thulasimani M Ramaswamy S. Artificial sweeteners as a sugar substitute: are they really safe? Indian J Pharmacol. (2016) 48:237. doi: 10.4103/0253-7613.182888 27298490 PMC4899993

[16] Nettleton JE Cho NA Klancic T Nicolucci AC Shearer J Borgland SL. Maternal low-dose aspartame and stevia consumption with an obesogenic diet alters metabolism, gut microbiota and mesolimbic reward system in rat dams and their offspring. Gut. (2020) 69:1807–17. doi: 10.1136/gutjnl-2018-317505, PMID: 31996393 PMC7497576

[17] Hall KD Ayuketah A Brychta R Cai H Cassimatis T Chen KY. Ultra-processed diets cause excess calorie intake and weight gain: an inpatient randomized controlled trial of ad libitum food intake. Cell Metab. (2019) 30:67–77.e3. doi: 10.1016/j.cmet.2019.05.008, PMID: 31105044 PMC7946062

[18] Lowery CM Mora-Plazas M Gómez LF Popkin B Taillie LS. Reformulation of packaged foods and beverages in the Colombian food supply. Nutrients. (2020) 12:3260. doi: 10.3390/nu12113260 33114419 PMC7692620

[19] Euromonitor International Limited. Euromonitor. Available at: http://www.euromonitor.com

[20] Harris PA Taylor R Thielke R Payne J Gonzalez N Conde JG. Research electronic data capture (REDCap)—a metadata-driven methodology and workflow process for providing translational research informatics support. J Biomed Inform. (2009) 42:377–81. doi: 10.1016/j.jbi.2008.08.010 18929686 PMC2700030

[21] Kanter R Reyes M Corvalán C. Photographic methods for measuring packaged food and beverage products in supermarkets. Curr Dev Nutr. (2017) 1:e001016. doi: 10.3945/cdn.117.001016 29955678 PMC5998779

[22] Mora-Plazas M Gómez LF Miles DR Parra DC Taillie LS. Nutrition quality of packaged foods in Bogotá, Colombia: a comparison of two nutrient profile models. Nutrients. (2019) 11:1011. doi: 10.3390/nu11051011 31060219 PMC6567873

[23] Pan American Health Organization. Nutrient profile model. Washington DC, USA: Pan American Health Organization (2016).

[24] Saavedra-Garcia L Meza-Hernández M Diez-Canseco F Taillie LS. Reformulation of top-selling processed and ultra-processed foods and beverages in the Peruvian food supply after front-of-package warning label policy. Int J Environ Res Public Health. (2022) 20:424. doi: 10.3390/ijerph20010424 36612748 PMC9819345

[25] Zancheta Ricardo C Corvalán C Smith Taillie L Quitral V Reyes M. Changes in the use of non-nutritive sweeteners in the Chilean food and beverage supply after the implementation of the food labeling and advertising law. Front Nutr. (2021) 8:773450. doi: 10.3389/fnut.2021.773450 34859036 PMC8630583

[26] Campbell N Browne S Claudy M Reilly K Finucane FM. Ultra-processed food: the tragedy of the biological commons. Int J Heal Policy Manag. (2023) 12:1–4. doi: 10.34172/ijhpm.2022.7557 PMC10125221 37579452

[27] Dicken SJ Batterham RL. Ultra-processed food and obesity: what is the evidence? Curr Nutr Rep. (2024) 13:23–38. doi: 10.1007/s13668-024-00517-z 38294671 PMC10924027

